# Objectification of Skin Surface Evenness: In Vivo Evaluation of 300 Women in Relation to Age

**DOI:** 10.1111/jocd.70383

**Published:** 2025-08-23

**Authors:** Alena Roessle, Martina Kerscher

**Affiliations:** ^1^ Cosmetic Science, Institute of Biochemistry and Molecular Biology University of Hamburg Hamburg Germany

**Keywords:** emergent perceptual categories, reference ranges, SELS parameters, skin quality, skin surface evenness, topography, Visioscan

## Abstract

**Background:**

Skin surface evenness is one of the four emergent perceptual categories (EPCs) used to assess skin quality (SQ) in clinical and aesthetic practice. While objective measurements are essential for evaluating age‐related changes and treatment outcomes, robust, site‐specific baseline data remain unavailable.

**Aim:**

This study aimed to establish the first normative reference ranges for skin surface evenness parameters in a large cohort of women, enabling objective comparison across anatomical sites and future benchmarking for individualized aesthetic treatment planning.

**Patients/Methods:**

A total of 300 Caucasian women aged 20–69 years were assessed using the Visioscan VC 20plus at the forehead, cheek, neck, décolleté, and hand. Surface Evaluation of Living Skin (SELS) parameters—roughness (SEr), scaliness (SEsc), smoothness (SEsm), and wrinkles (SEw)—were analyzed. Pearson correlation coefficients were calculated to assess age‐related changes. Age‐stratified reference ranges were derived using percentile distributions.

**Results:**

Moderate positive correlations with age were observed for SEsm at the neck (*r* = 0.454, *p* < 0.01) and décolleté (*r* = 0.475, *p* < 0.01), and for SEw at the décolleté (*r* = 0.385, *p* < 0.05). Other parameters and regions exhibited low or negligible age correlations. Reference ranges for key parameters were defined across age groups.

**Conclusions:**

This study provides the first age‐based reference ranges for skin surface evenness in women, revealing site‐specific aging patterns most pronounced in the neck and décolleté. These baselines enable objective evaluation of treatment efficacy and support personalized aesthetic interventions. By complementing established baselines for other EPCs, such as skin firmness, these findings contribute to a unified framework for objectively assessing and tracking skin quality across the aging spectrum.

## 1 Introduction

Skin quality (SQ) is a foundational concept in aesthetic dermatology, defined by an international consensus through the four emergent perceptual categories (EPCs): skin surface evenness, skin tone evenness, skin firmness, and skin glow. These EPCs collectively shape how skin health and age are perceived, and their assessment is increasingly used to guide both clinical evaluations and aesthetic dermatology [1].

Among the EPCs, skin surface evenness plays a particularly central role in the perception of age [2, 3]. This dimension reflects a complex interplay of intrinsic factors, such as genetic predisposition and cellular aging, and extrinsic stressors, including ultraviolet (UV) radiation, environmental exposure, and lifestyle choices [4]. Aging triggers structural and functional changes across all layers of the skin, including degeneration of collagen and elastin networks, directly impacting the biomechanical properties of the skin [5], as previously quantified by Roessle and Kerscher in the context of skin firmness [6]. In parallel, skeletal remodeling during aging, such as bone resorption and ligament laxity, alters facial topography and further contributes to surface unevenness. The ultimate reduction of structural support promotes volume loss and fat redistribution, leading to visible contour changes [7].

Skin surface evenness is shaped by six perceptual parameters: pores, crepiness, wrinkles and lines, (acne) scars, hair, and clarity [1]. Of these, wrinkles/lines and crepiness can be objectively quantified using the Visioscan system through derived Surface Evaluation of Living Skin (SELS) parameters, including roughness (SEr), scaliness (SEsc), smoothness (SEsm), and wrinkles (SEw). Despite its relevance, objective, age‐based baseline data for skin surface evenness are lacking. Previous studies suffer from small sample sizes, heterogenous methods, and limited anatomical coverage, including methodological heterogeneity, small sample sizes, and a restricted focus on specific body sites [8, 9]. These constraints limit the generalizability of their findings and underscore the need for larger, systematically structured datasets to enable robust, age‐stratified analyses, and benchmarking.

Moreover, the subjective perception of surface irregularities, such as wrinkles or scarring, varies widely between patients and clinicians, potentially affecting treatment motivation and satisfaction. By contrast, objective measurements offer reproducible benchmarks that can be used to track progress, guide interventions, and compare outcomes.

While reference ranges for skin firmness have already been established [6] no equivalent data currently exist for skin surface evenness. This represents a critical gap in the development of a holistic, measurable approach to SQ.

This study aims to establish the first age‐stratified reference ranges for skin surface evenness in a large cohort of women across five anatomical sites. In doing so, it complements earlier work on another EPC, skin firmness, and offers a new tool to support personalized, data‐driven aesthetic care, both in the selection of interventions and in the evaluation of treatment effects.

## 2 Materials and Methods

The Ethics Committee of Hamburg, Ethik‐Kommission der Ärztekammer Hamburg (2022–100 840‐BO‐ff) approved this study and conducted it in accordance with scientific standards and ethical guidelines as detailed in Roessle and Kerscher [6]. Between September 2022 and March 2024, a total of 300 Caucasian women (20–69 years) were recruited, following the same inclusion and exclusion criteria as described in Roessle and Kerscher [6] and shown in Table 1. Written informed consent was obtained from all subjects prior to the start of the study. Participants were divided into five cohorts age groups (AG) of 60 subjects: AG I (20–29 years), AG II (30–39 years), AG III (40–49 years), AG IV (50–59 years), and AG V (60–69 years).

**TABLE 1:** Eligibility criteria as Roessle and Kerscher [6].

Inclusion criteria	Caucasians with Fitzpatrick skin type (FST) I–IV
	Age requirements 20–49 years: Premenopausal In the follicular phase of the menstrual cycle No recent changes in hormonal contraception (within the last 3 months) 50+ years: Postmenopausal No hormone replacement therapy for at least 1 year
	Consistent skin care routine for a minimum of 3 months
	Unchanged skin care routine for at least 3 months
	Healthy skin and overall stable health condition
Exclusion criteria	Body Mass Index under 18.5 or 30 and above
	Factors affecting the measurement area (e.g., scars, tattoos)
	History or current signs of tanorexia or severe sunburn
	Skin aging beyond typical expectations for the participant's age
	Pregnancy or breastfeeding
	High nicotine intake (≥ 20 cigarettes per week for over 2 years)
	Substance abuse, including excessive alcohol or drugs
	Any condition or treatment that could alter skin appearance: Phototherapy or chemotherapy within the past 6 months. Use of prescription oral or topical anti‐aging/skin improvement products in the last 3 months. Immunosuppressive or immunomodulatory medications, including systemic corticosteroids. Cosmetic or medical skin treatments in the previous 3 months. Any (minimally) invasive procedure at the measurement sites.
	Smoking or caffeine consumption before or during the visit
	Application of topical products within 12 h prior to the visit
	Water contact on the measurement sites within 6 h before the visit
	Exposure to extreme temperatures (e.g., cryotherapy, sauna)
	Exercise or alcohol consumption within 24 h prior to the visit
	Participation in another clinical study within the past 30 days

**FIGURE 1:**
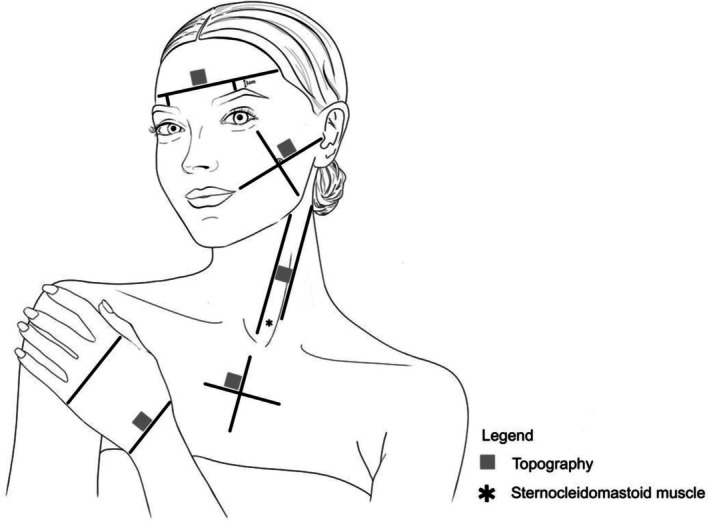
Skin areas for Visioscan adapted from Roessle and Kerscher [6].

One skin surface topography measurement was performed at each of the five different areas (forehead, cheek, neck, décolleté, and dorsum of the hand; Figure 1) under standardized conditions (30‐min acclimatization, 20°C–22°C with 40%–60% relative humidity) as in Roessle and Kerscher [6]. The side (right/left) of measurement was randomized per participant.

The Visioscan VC 20plus system (Courage+Khazaka electronic GmbH, Cologne, Germany) was used to assess skin surface parameters by capturing images that encode height and depth variations into gray values. Image analysis software (Visioscan FW) calculated four SELS skin parameters: SEr, SEsc, SEsm, and SEw. Lower SEr values indicate higher roughness, lower SEsc values suggest less desquamation, lower SEsm values denote higher smoothness, and lower SEw values reflect fewer wrinkles [10]. The measurement results are dependent on the calculation area, which is why size 2 as measured area is selected in accordance with the manufacturer's recommendations to prevent possible artifacts.

**FIGURE 2:**
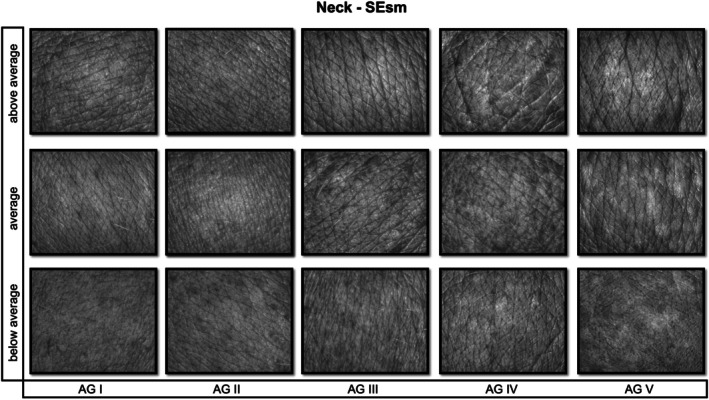
Visioscan images illustrating skin smoothness (SEsm) on the neck, shown for below‐average, average, and above‐average values within each age group (I–V). Lower SEsm values denote higher smoothness [10].

**FIGURE 3:**
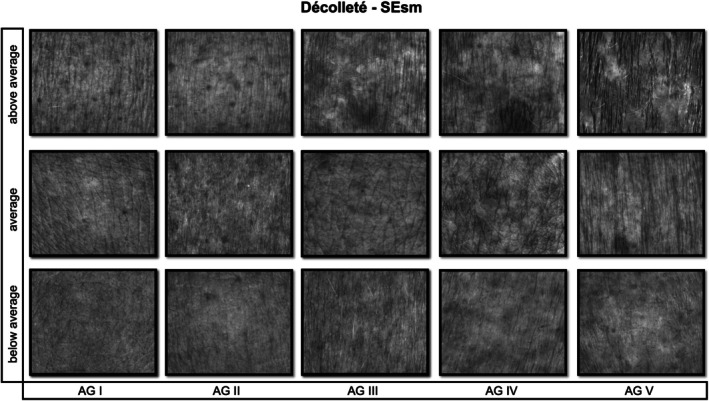
Visioscan images illustrating skin smoothness (SEsm) on the décolleté, shown for below‐average, average, and above‐average values within each age group (I–V). Lower SEsm values denote higher smoothness [10].

**FIGURE 4:**
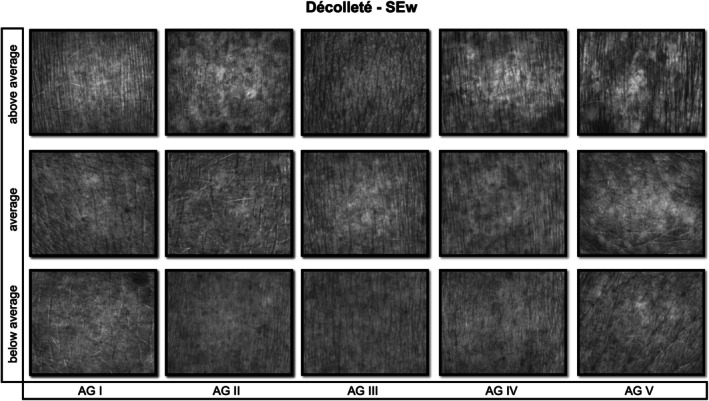
Visioscan images illustrating skin wrinkles (SEw) on the décolleté, shown for below‐average, average, and above‐average values within each age group (I–V). Lower SEw values reflect fewer wrinkles [10].

As described in Roessle and Kerscher, the statistical analysis was conducted [6]. Using SPSS software (Version 26, IBM Corp., Armonk, NY, USA) the Bravais–Pearson test was performed. Pearson's correlation coefficients (*r*) were used to determine the linear associations between age and each SELS parameter and interpreted according to Cohen's definitions: low (*r* ≥ 0.1), moderate (*r* ≥ 0.3), and high (*r* ≥ 0.5), degree of correlation (positive or negative) and significance (*p* ≤ 0.05). To establish reference ranges, the first (25th) and third (75th) percentiles of parameters with a moderate Pearson correlation coefficient were selected. The reference ranges were categorized as below average, average, and above average [6]. Figures 2, 3, 4 provide illustrative Visioscan images for key parameters exemplifying each reference range across the defined age groups.

## 3 Results

The laboratory conditions (20.35°C ± 1.52°C, relative humidity 51.85% ± 6%) during the measurement period are the same as described in Roessle and Kerscher, as is the distribution of demographic data for the 300 participants [6].

**FIGURE 5:**
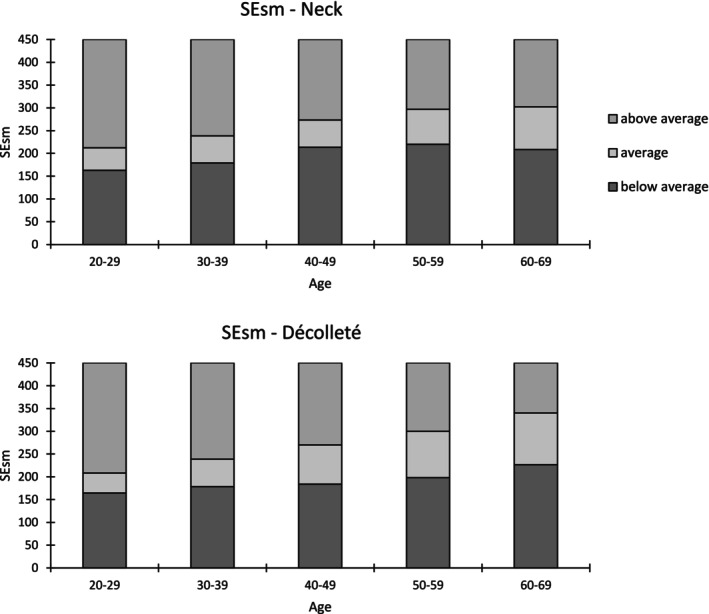
Reference ranges of SEsm for the neck, and décolleté.

**FIGURE 6:**
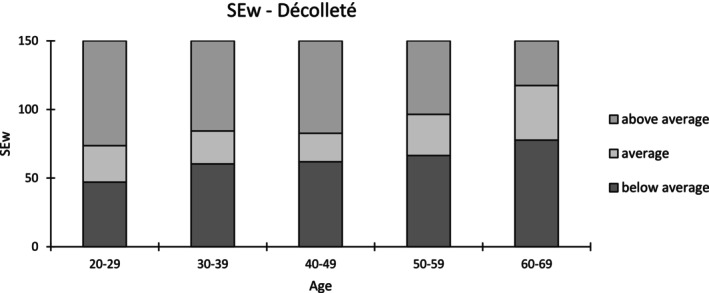
Reference ranges of SEw for the décolleté.

Table S1 presents the mean values, their standard deviations (SDs), and the corresponding percentiles. The correlation coefficients for Surface Evaluation of Living Skin (SELS) parameters with age areas across different anatomical sites are shown in Table 2. The reference ranges are presented graphically in Figures 5 and 6.

**TABLE 2:** Correlation coefficients of the SELS parameters and age per skin area.

SELS parameters	Forehead	Cheek	Neck	Décolleté	Dorsum of the hand
SEr	−0.140*	−0.126*	0.032	−0.114*	0.268*
SEsc	−0.021	0.195**	0.273**	0.241**	0.212**
SEsm	0.088	0.056	0.454**	0.475**	0.275**
SEw	−0.136*	0.033	0.272**	0.385*	0.260*

* *p* < 0.05.

** *p* < 0.01.

### 3.1 Forehead

The forehead exhibited low negative correlations with age for SEr (*r* = −0.140, *p* < 0.05) and SEw (*r* = −0.136, *p* < 0.05), while SEsc (*r* = −0.021, *p* > 0.05) and SEsm (*r* = 0.088, *p* > 0.05) had correlations with age close to zero.

### 3.2 Cheek

On the cheek, SEr (*r* = −0.126, *p* < 0.05) showed a low negative correlation, while SEsc (*r* = 0.195, *p* < 0.01) exhibited a significant low positive correlation. The parameters SEsm (*r* = 0.056, *p* > 0.05) and SEw (*r* = 0.033, *p* > 0.05) had correlations with age close to zero.

### 3.3 Neck

The parameter SEsm (*r* = 0.454, *p* < 0.01) exhibited a significantly moderate positive correlation with age. A significant low positive correlation with age was found for SEsc (*r* = 0.273, *p* < 0.01) and SEw (*r* = 0.272, *p* < 0.01). SEr (*r* = 0.032, *p* > 0.05) showed a correlation with age close to zero.

### 3.4 Décolleté

The parameter SEsm (*r* = 0.475, *p* < 0.01) and SEw (*r* = 0.385, *p* < 0.05) exhibited a significantly moderate positive correlations with age. With a negative correlation coefficient for SEr (*r* = −0.114, *p* < 0.05) and a positive one for SEsc (*r* = 0.241, *p* < 0.01), significantly low correlations with age were found.

### 3.5 Hand

SEr (*r* = 0.268, *p* < 0.05), SEsc (*r* = 0.212, *p* < 0.01), SEsm (*r* = 0.275, *p* < 0.01), and SEw (*r* = 0.260, *p* < 0.05) all demonstrated a statistically significant, though low, positive correlation with age.

## 4 Discussion

This study investigated age‐related skin surface evenness across five anatomical sites in a large cohort of 300 women using Visioscan‐derived SELS parameters. These parameters—SEr, SEsc, SEsm, and SEw—offer objective quantification of skin topography and were used to establish the first age‐stratified reference ranges for this EPC.

Of the four SELS parameters, SEsm and Sew exhibited moderate correlations with in site‐specific patterns: SEsm increased with age at the neck (*r* = 0.454, *p* < 0.01) and décolleté (*r* = 0.475, *p* < 0.01), while SEw also increased at the décolleté (*r* = 0.385, *p* < 0.05). These findings reflect a decline in smoothness and an increase in wrinkle formation with age, consistent with structural degradation in the regions.

In contrast, the face (forehead, cheek) showed only low or negligible correlations despite being regularly exposed to extrinsic aging factors such as UV radiation and pollution [4, 5]. This counterintuitive result may be explained by the widespread use of preventive skincare, cosmetic treatments, and sun protection in facial regions—interventions that may mask chronological changes in surface evenness [11, 12]. Importantly, subjects with aesthetic procedures were excluded, but the long‐term preventive effects of daily care may still influence surface metrics.

Compared to earlier studies, our results offer a more detailed anatomical perspective. Trojahn et al. (*N* = 38), examining a heterogeneous cohort including children (3.6 ± 1.4 years), young adults (32.9 ± 7.2 years), and older adults (68.3 ± 2.5 years), found positive correlations between SEsm (*r* = 0.558) and SEr (*r* = 0.379) with age on the volar forearm. Weak correlations were observed for SEw and SEsc [8]. These findings only partially align with our results, likely due to population heterogeneity and limited body site analysis. Similarly, Sanabria‐de la Torre et al. (*N* = 44, 29 females/14 males; mean age 38.8 ± 15 years) using the Visioscan VC 20plus found no significant difference in SELS parameters by age, at the forearm, cheek, or palm [9]. These underscore the importance of anatomical specificity and large sample size to detect subtle topographic changes.

This study addresses a key gap in the literature by providing baseline values for skin surface evenness, complementing earlier work on EPC skin firmness [6]. These reference ranges can serve as objective benchmarks for monitoring aging, evaluating treatment outcomes, and supporting individualized aesthetic planning. The neck and décolleté, in particular, emerged as reliable regions for age‐related topographic assessment and may warrant greater focus in rejuvenation strategies.

Our findings align with previous treatment‐focused studies by Kerscher et al., which demonstrated improvements in skin roughness (Rz) on the lower cheeks following multiple‐dose treatment with CPM‐HA20G (a cohesive polydensified matrix hyaluronic acid with glycerol; Belotero Revive, Anteis S.A., Lonay, Switzerland; a Merz Aesthetics group company) at 16 and 32 weeks [13]. The present data also support a broader framework for evaluating EPC changes before and after intervention. Microneedling, chemical peels, and injectable therapies all represent viable tools for improving surface evenness and could be evaluated against these newly established norms. Topical treatments may also be effective [1].

Despite its strengths and novelty, this study has limitations. The sample, though large (*N* = 300) was limited to healthy Caucasian women, providing a strong baseline but restricting generalizability to a more diverse population. Not all parameters contributing to skin surface evenness, as defined by Goldie et al., could be measured objectively; for instance, pore size and clarity were not assessed, as they require 3D imaging systems such as LifeViz [1]. Additionally, the Visioscan system does not fully address parameters such as scarring or hair presence, which may affect surface perception. Lastly, lifestyle factors (e.g., sun exposure, skincare habits) were not considered.

In conclusion, only three moderate correlations between SELS parameters and age were detected, suggesting that SEsm and SEw at the neck and décolleté are the most sensitive markers of age‐related surface change, as measured by surface level topography. Other SELS parameters showed limited correlation, indicating that while useful, these measures alone may not fully capture the complexity of aging skin. Future research should focus on exploring alternative markers or complementary tools, integrating broader, diverse populations, and continuing to assess and integrate other EPCs to advance objective, holistic skin quality evaluation.

## Author Contributions

The author, Alena Roessle, is responsible for the conception, design, data collection, data analysis, and writing of this manuscript. Prof. Kerscher provided guidance and advisory support throughout the study process.

## Ethics Statement

The authors confirm that the ethical policies of the journal, as outlined on the journal's author guidelines page, have been adhered to, and appropriate approval from the ethical review committee has been obtained. The Independent Ethics Committee (Ethikkommission der Ärztekammer Hamburg) in Germany approved the study (2022–100 840‐BO‐ff) on September 20, 2022. The study was conducted in accordance with the principles of the Declaration of Helsinki and the International Conference on Harmonization Guidelines for Good Clinical Practice. Measurements were not taken prior to the signing of the informed consent.

## Conflicts of Interest

The authors declare no conflicts of interest.

## Supporting information


**Table S1.** Mean ± SD and median with quartiles (Q_1_, Q_3_) of the SELS parameters per site and age group.

## Data Availability

The data that support the findings of this study are available from the corresponding author upon reasonable request.

## References

[1] K. Goldie , M. Kerscher , S. G. Fabi , et al., “Skin Quality—A Holistic 360° View: Consensus Results,” CCID 14 (2021): 643–654, 10.2147/CCID.S309374. 34163203 PMC8214518

[2] N. Samson , B. Fink , P. J. Matts , N. C. Dawes , and S. Weitz , “Visible Changes of Female Facial Skin Surface Topography in Relation to Age and Attractiveness Perception: Perception of Facial Skin Surface Topography,” Journal of Cosmetic Dermatology 9, no. 2 (2010): 79–88, 10.1111/j.1473-2165.2010.00489.x. 20618552

[3] N. Samson , B. Fink , and P. Matts , “Interaction of Skin Color Distribution and Skin Surface Topography Cues in the Perception of Female Facial Age and Health: Perception of Skin Color and Topography,” Journal of Cosmetic Dermatology 10, no. 1 (2011): 78–84, 10.1111/j.1473-2165.2010.00538.x. 21332921

[4] J. Krutmann , A. Bouloc , G. Sore , B. A. Bernard , and T. Passeron , “The Skin Aging Exposome,” Journal of Dermatological Science 85, no. 3 (2017): 152–161, 10.1016/j.jdermsci.2016.09.015. 27720464

[5] T. Quan , “Molecular Insights of Human Skin Epidermal and Dermal Aging,” Journal of Dermatological Science 112, no. 2 (2023): 48–53, 10.1016/j.jdermsci.2023.08.006. 37661473 PMC13155249

[6] A. Roessle and M. Kerscher , “Objectification of Skin Firmness: In Vivo Evaluation of 300 Women in Relation to Age,” Journal of Cosmetic Dermatology 24, no. 1 (2025): e16773, 10.1111/jocd.16773. 39780520 PMC11712028

[7] S. Cotofana , A. Fratila , T. Schenck , W. Redka‐Swoboda , I. Zilinsky , and T. Pavicic , “The Anatomy of the Aging Face: A Review,” Facial Plastic Surgery 32, no. 3 (2016): 253–260, 10.1055/s-0036-1582234. 27248022

[8] C. Trojahn , G. Dobos , M. Schario , L. Ludriksone , U. Blume‐Peytavi , and J. Kottner , “Relation Between Skin Micro‐Topography, Roughness, and Skin Age,” Skin Research and Technology 21, no. 1 (2015): 69–75, 10.1111/srt.12158. 24889351

[9] R. Sanabria‐de La Torre , M. Ceres‐Muñoz , C. Pretel‐Lara , T. Montero‐Vílchez , and S. Arias‐Santiago , “Microtopography and Barrier Function in Healthy Skin: Differences Between Forearm, Cheek and Palm,” Cosmetics 11, no. 1 (2023): 5, 10.3390/cosmetics11010005.

[10] H. Tronnier , M. Wiebusch , U. Heinrich , and R. Stute , “Surface Evaluation of Living Skin,” Advances in Experimental Medicine and Biology 455 (1999): 507–516, 10.1007/978-1-4615-4857-7_75. 10599390

[11] Z. D. Draelos , L. Wei , M. Sachdev , et al., “International Consensus on Anti‐Aging Dermocosmetics and Skin Care for Clinical Practice Using the RAND/UCLA Appropriateness Method,” JDD 23, no. 1 (2024): 1337–1343, 10.36849/JDD.7798. 38206152

[12] A. J. Oh and D. B. Rootman , “Recent Fluctuations in Public Searches for Cosmetic Procedures as Shown by Google Trends,” Ophthalmic Plastic and Reconstructive Surgery 40 (2023): 266–269, 10.1097/IOP.0000000000002562. 37972973

[13] M. Kerscher , W. Prager , T. C. Fischer , et al., “Facial Skin Revitalization With Cohesive Polydensified Matrix‐HA20G: Results From a Randomized Multicenter Clinical Study,” Plastic and Reconstructive Surgery. Global Open 9, no. 12 (2021): e3973, 10.1097/GOX.0000000000003973. 35070607 PMC8769088

