# Supplementary material for: Objectification of Skin Surface Evenness: In Vivo Evaluation of 300 Women in Relation to Age
Source: J Cosmet Dermatol. 2025 Aug 23;24(Suppl 4):e70383. doi: 10.1111/jocd.70383 (PMC12374563; doi:10.1111/jocd.70383)
Supplement: Supplementary file 1 — Table S1. Mean ± SD and median with quartiles (Q1, Q3) of the SELS parameters per site and age group. [file JOCD-24-e70383-s001.docx]

Table S1: Mean ± SD and median with quartiles (Q_1_, Q_3_) of the SELS parameters per site and age group.

| **SELS parameters** | **Sites** | | | | |
| --- | --- | --- | --- | --- | --- |
|  | **Forehead** | **Cheek** | **Neck** | **Décolleté** | **Hand** |
| **SEr** | | | | | |
| AG I  n=60 | 5.27±2.13  Q_1_=3.68  Med=4.78  Q_3_=6.31 | 4.06±1.52  Q_1_=3.012  Med=3.68  Q_3_=4.82 | 2.00±0.97  Q_1_=1.32  Med=1.92  Q_3_=2.30 | 3.24±1.55  Q_1_=2.17  Med=2.86  Q_3_=3.86 | 2.83±0.998  Q_1_=2.28  Med=2.77  Q_3_=3.44 |
| AG II  n=60 | 5.24±2.07  Q_1_=3.40  Med=4.60  Q_3_=6.62 | 3.59±1.22  Q_1_=2.50  Med=3.34  Q_3_=4.40 | 2.02±0.70  Q_1_=1.49  Med=1.91  Q_3_=2.48 | 3.52±1.37  Q_1_=2.64  Med=3.16  Q_3_=3.93 | 3.26±1.19  Q_1_=2.26  Med=3.17  Q_3_=3.91 |
| AG III  n=60 | 4.78±1.76  Q_1_=3.41  Med=4.39  Q_3_=5.69 | 3.95±1.58  Q_1_=3.06  Med=3.59  Q_3_=4.23 | 2.17±1.12  Q_1_=1.43  Med=1.97  Q_3_=2.64 | 3.11±1.25  Q_1_=2.30  Med=2.96  Q_3_=3.78 | 3.54±1.06  Q_1_=2.96  Med=3.52  Q_3_=4.13 |
| AG IV  n=60 | 4.50±1.49  Q_1_=3.44  Med=4.14  Q_3_=5.11 | 3.48±1.03  Q_1_=2.68  Med=3.40  Q_3_=3.93 | 1.95±0.71  Q_1_=1.49  Med=1.83  Q_3_=2.32 | 2.84±0.98  Q_1_=2.31  Med=2.79  Q_3_=3.40 | 3.70±1.23  Q_1_=2.88  Med=3.45  Q_3_=4.59 |
| AG V  n=60 | 4.82±1.73  Q_1_=3.64  Med=4.20  Q_3_=5.65 | 3.56±1.04  Q_1_=2.73  Med=3.58  Q_3_=4.11 | 2.12±0.80  Q_1_=1.57  Med=1.97  Q_3_=2.39 | 3.09±0.97  Q_1_=2.52  Med=2.99  Q_3_=3.47 | 3.76±1.31  Q_1_=2.91  Med=3.45  Q_3_=4.57 |
| **SEsc** | | | | | |
| AG I  n=60 | 0.10±0.16  Q_1_=0.01  Med=0.02  Q_3_=0.09 | 0.17±0.24  Q_1_=0.02  Med=0.06  Q_3_=0.22 | 0.38±0.49  Q_1_=0.05  Med=0.18  Q_3_=0.55 | 0.19±0.38  Q_1_=0.01  Med=0.07  Q_3_=0.17 | 0.22±0.38  Q_1_=0.02  Med=0.05  Q_3_=0.24 |
| AG II  n=60 | 0.14±0.20  Q_1_=0.02  Med=0.06  Q_3_=0.15 | 0.38±0.42  Q_1_=0.08  Med=0.24  Q_3_=0.49 | 0.37±0.39  Q_1_=0.09  Med=0.23  Q_3_=0.57 | 0.15±0.19  Q_1_=0.01  Med=0.08  Q_3_=0.23 | 0.12±0.16  Q_1_=0.01  Med=0.05  Q_3_=0.17 |
| AG III  n=60 | 0.22±0.40  Q_1_=0.02  Med=0.07  Q_3_=0.18 | 0.53±0.52  Q_1_=0.13  Med=0.40  Q_3_=0.79 | 0.78±0.90  Q_1_=0.17  Med=0.39  Q_3_=0.86 | 0.49±0.68  Q_1_=0.04  Med=0.18  Q_3_=0.73 | 0.36±0.72  Q_1_=0.04  Med=0.13  Q_3_=0.35 |
| AG IV  n=60 | 0.11±0.11  Q_1_=0.03  Med=0.09  Q_3_=0.14 | 0.60±0.68  Q_1_=0.13  Med=0.34  Q_3_=0.83 | 0.99±0.81  Q_1_=0.37  Med=0.85  Q_3_=1.26 | 0.67±0.77  Q_1_=0.09  Med=0.42  Q_3_=1.04 | 0.53±0.68  Q_1_=0.11  Med=0.29  Q_3_=0.81 |
| AG V  n=60 | 0.11±0.22  Q_1_=0.01  Med=0.04  Q_3_=0.13 | 0.45±0.67  Q_1_=0.12  Med=0.25  Q_3_=0.48 | 0.77±0.67  Q_1_=0.30  Med=0.60  Q_3_=1.04 | 0.43±0.56  Q_1_=0.12  Med=0.23  Q_3_=0.61 | 0.45±0.61  Q_1_=0.05  Med=0.22  Q_3_=0.57 |
| **SEsm** | | | | | |
| AG I  n=60 | 198.08±68.37  Q_1_=150.43  Med=181.56  Q_3_=223.09 | 228.05±72.69  Q_1_=184.10  Med=207.79  Q_3_=263.49 | 190.61±36.75  Q_1_=163.15  Med=187.97  Q_3_=212.39 | 187.94±39.18  Q_1_=164.68  Med=184.58  Q_3_=208.49 | 182.01±32.63  Q_1_=156.94  Med=178.07  Q_3_=202.81 |
| AG II  n=60 | 253.18±79.62  Q_1_=191.07  Med=245.75  Q_3_=294.61 | 301.60±131.96  Q_1_=210.31  Med=260.31  Q_3_=356.90 | 211.75±47.62  Q_1_=179.13  Med=210.58  Q_3_=238.36 | 210.71±44.39  Q_1_=178.79  Med=200.67  Q_3_=238.97 | 202.99±49.72  Q_1_=168.32  Med=192.00  Q_3_=230.97 |
| AG III  n=60 | 236.10 ±77.31  Q_1_=185.35  Med=220.28  Q_3_=272.89 | 256.02±70.51  Q_1_=199.27  Med=239.83  Q_3_=307.56 | 244.96±45.70  Q_1_=213.52  Med=243.44  Q_3_=273.46 | 234.73±63.86  Q_1_=184.14  Med=220.74  Q_3_=270.27 | 228.18±83.10  Q_1_=171.69  Med=203.31  Q_3_=256.74 |
| AG IV  n=60 | 237.46±85.44  Q_1_=175.60  Med=217.03  Q_3_=270.18 | 251.65±76.75  Q_1_=198.34  Med=240.30  Q_3_=286.14 | 255.45±47.67  Q_1_=220.28  Med=249.03  Q_3_=296.83 | 258.02±78.54  Q_1_=198.05  Med=243.49  Q_3_=299.99 | 226.00±62.74  Q_1_=181.94  Med=228.52  Q_3_=245.22 |
| AG V  n=60 | 238.95±87.41  Q_1_=187.54  Med=222.34  Q_3_=275.59 | 283.18±90.73  Q_1_=213.01  Med=254.68  Q_3_=342.75 | 260.91±66.93  Q_1_=208.54  Med=244.96  Q_3_=302.33 | 284.62±71.17  Q_1_=226.88  Med=271.55  Q_3_=340.15 | 236.42±67.67  Q_1_=185.25  Med=238.16  Q_3_=287.26 |
| **SEw** | | | | | |
| AG I  n=60 | 79.91±35.18  Q_1_=58.36  Med=72.13  Q_3_=89.31 | 81.05±29.25  Q_1_=58.92  Med=80.15  Q_3_=91.13 | 51.53±12.74  Q_1_=42.93  Med=47.91  Q_3_=58.74 | 61.84±17.31  Q_1_=47.70  Med=60.19  Q_3_=73.70 | 67.76±19.56  Q_1_=54.16  Med=62.89  Q_3_=77.00 |
| AG II  n=60 | 105.90±36.90  Q_1_=76.24  Med=103.71  Q_3_=125.27 | 105.34±44.32  Q_1_=73.73  Med=97.41  Q_3_=123.93 | 59.51±12.52  Q_1_=50.50  Med=57.16  Q_3_=65.65 | 75.69±20.85  Q_1_=60.36  Med=72.86  Q_3_=84.31 | 89.03±29.01  Q_1_=66.48  Med=81.52  Q_3_=104.81 |
| AG III  n=60 | 90.83±31.57  Q_1_=65.93  Med=89.27  Q_3_=104.88 | 81.88±24.21  Q_1_=62.35  Med=80.96  Q_3_=93.62 | 61.60±12.41  Q_1_=53.47  Med=58.80  Q_3_=72.01 | 78.83±30.62  Q_1_=61.86  Med=72.01  Q_3_=82.61 | 94.00±43.47  Q_1_=72.06  Med=82.25  Q_3_=106.22 |
| AG IV  n=60 | 99.79±43.45  Q_1_=68.85  Med=89.02  Q_3_=117.38 | 79.29±20.96  Q_1_=64.48  Med=77.28  Q_3_=92.89 | 60.62±13.78  Q_1_=51.53  Med=60.72  Q_3_=72.13 | 82.51±25.91  Q_1_=66.53  Med=79.02  Q_3_=96.46 | 90.14±31.17  Q_1_=68.45  Med=90.52  Q_3_=105.14 |
| AG V  n=60 | 104.89±40.05  Q_1_=80.01  Med=98.24  Q_3_=117.39 | 102.44±33.79  Q_1_=75.79  Med=92.89  Q_3_=121.79 | 65.24±17.18  Q_1_=53.30  Med=60.84  Q_3_=74.06 | 97.01±29.15  Q_1_=77.69  Med=92.09  Q_3_=117.46 | 101.46±36.39  Q_1_=75.03  Med=97.91  Q_3_=123.41 |

Q_1_=25th percentile, Q_3_=75^th^ percentile
